# Moist Heat or Dry Heat for Delayed Onset Muscle Soreness

**DOI:** 10.4021/jocmr1521w

**Published:** 2013-10-12

**Authors:** Jerrold Petrofsky, Lee Berk, Gurinder Bains, Iman Akef Khowailed, Timothy Hui, Michael Granado, Mike Laymon, Haneul Lee

**Affiliations:** a Department of Physical Therapy, Loma Linda University, Loma Linda, California, USA

**Keywords:** Heat, Moist, Muscle damage, Pain

## Abstract

**Background:**

Heat is commonly used in physical therapy following exercise induced delayed onset muscle soreness (DOMS). Most heat modalities used in a clinical setting for DOMS are only applied for 5 to 20 minutes. This minimal heat exposure causes little, if any, change in deep tissue temperature. For this reason, long duration dry chemical heat packs are used at home to slowly and safely warm tissue and reduce potential heat damage while reducing pain associated from DOMS. Clinically, it has been shown that moist heat penetrates deep tissue faster than dry heat. Therefore, in home use chemical moist heat may be more efficacious than dry heat to provide pain relief and reduce tissue damage following exercise DOMS. However, chemical moist heat only lasts for 2 hours compared to the 8 hours duration of chemical dry heat packs. The purpose of this study was to compare the beneficial effect of dry heat versus moist heat on 100 young subjects after exercise induce DOMS.

**Methods:**

One hundred subjects exercised for 15 minutes accomplishing squats. Before and for 3 days after, strength, muscle soreness, tissue resistance, and the force to passively move the knee were recorded. Heat and moist heat were applied in different groups either immediately after exercise or 24 hours later.

**Results:**

The research results of this study showed that immediate application of heat, either dry (8 hours application) or moist (2 hours application), had a similar preservation of quadriceps muscle strength and muscle activity. Results also revealed that the greatest pain reduction was shown after immediate application of moist heat. Never the less, immediate application of dry heat had a similar effect but to a lesser extent.

**Conclusion:**

It should be noted that moist heat had not only similar benefits of dry heat but in some cases enhanced benefits, and with only 25% of the time of application of the dry heat.

## Introduction

Heat has been used therapeutically for thousands of years [1]. It offers immediate pain relief and can increase circulation to speed the healing process after injury [2-4]. For this reason, it is popular for use on many types of pain including joint and muscle pain as well as soft tissue damage.

The effect of heat on pain is mediated by heat sensitive calcium channels [5]. These channels respond to heat by increasing intracellular calcium [6, 7]. This, in itself, generates action potentials that increases stimulation of sensory nerves and causes the feeling of heat in the brain [8]. These channels are part of a family of receptors called TRPV receptors [7]. TRPV1 and TRPV2 channels are sensitive to noxious heat [7], while TRPV4 channels are sensitive to normal physiological heat [7, 9]. These channels have in common their sensitivity to other substances such as vanilla and menthol [10]. These multiple binding sites allow a number of factors to activate these channels. Once activated, they can also inhibit the activity of purine pain receptors [11]. These receptors, termed P2X2 and P2Y2 receptors, are mediated pain receptors and are located in the peripheral small nerve endings [11]. For peripheral pain, for example, heat can directly inhibit pain [12]. However, when pain is originating from deep tissue, heat stimulates peripheral pain receptors which can alter what has been termed gating in the spinal cord and reduce deep pain [13].

Another effect of heat is its ability to increase circulation [14-18]. These same TRPV1 and TRPV4 receptors along with noiciceptors, increase blood flow in response to heat. The initial response to heat is mediated through sensory nerves that release substance P and calcitonin G related peptide to increase circulation [19-22]. After a minute or so, nitric oxide is produced in vascular endothelial cells and is responsible for the sustained response of the circulation to heat. This increase in circulation is considered to be essential in tissue protection from heat and repair of damaged tissue.

Heat is used in different modalities in the treatment of back pain and muscle soreness. Dry heat can be applied through either heat packs [2] or techniques that warm tissue such as diathermy and ultrasound [23-25]. Heat packs can be dry or moist. Hydrocolator heat packs are usually at 165 deg F and are separated from the skin by 6 - 8 layers of towels and used only in clinical settings [17]. Hydrotherapy (warm) uses water at 105 deg F and involves immersing a limb in the water. Hydrotherapy can include contrast baths or simply warm water immersion [17]. A major problem with this type of heating is that it is usually used for short periods of time, for example, 5 - 20 minutes. Moist heat, in most studies, appears to be advantageous in pain relief to many short duration dry heat modalities such as electric heat pads [15, 26]. But all of these heat modalities are used for short periods of time, for example, 20 minutes maximum. Many studies have shown that short duration of heat application results in poor heat transfer to deep tissues [3, 15, 17, 27]. Therefore, in deep injuries, heat application for short duration causes pain relief through the gate control theory of pain in the central nervous system and not through the peripheral nervous system. Long term application of heat, such as in chemical heat wraps, solves this issue by applying heat for hours to warm deep tissue gradually [28, 29]. But dry chemical heat wraps heat deep tissue much slower that pain relief is delayed by at least 30 minutes. A faster heating modality is chemical moist heat. Here, heat penetration is fast but the duration of chemical moist heat is usually between 30 minutes and 2 hours [15]. The question, then, is how moist and chemical dry heat compare for healing and pain relief. This study compared chemical moist heat lasting 2 hours to dry heat lasting 8 hours to distinguish which heat modality worked best on the symptoms of delayed onset muscle soreness.

### Subjects

The subjects in this study were 100 healthy individuals between the ages of 20 and 40 years old, divided randomly into 5 groups. The groups were 1), control; 2), moist heat immediately after exercise; 3), moist heat applied 24 hours after exercise; 4), dry heat pack applied immediately after exercise and 5) dry heat pack applied 24 hours after exercise. Their body mass index (BMI) was less than 40. Subjects had no cardiovascular disease, hepatic disease, diabetes, lower limb neuropathies, or recent lower limb injuries. Subjects were not taking alpha or beta agonist/antagonists, any type of NSAIDS, Cox 2 inhibitors, Calcium channel blockers, Pregabalins (Lyrica), or pain reducers. The demographics of the subjects are shown in Tables 1-5. There was no statistical difference between the ages, heights and weights of the groups. All methods and procedures were approved by the Institutional Review Board of Loma Linda University and all subjects signed a statement of informed consent.

**Table 1:** Demographics of Control Group

	Age (years)	Height (cm)	Weight (kg)	BMI
mean	25.3	165.9	63.7	23.1
sd	3.0	6.0	10.4	3.5

**Table 2:** Demographics of the Moist Heat Immediate After Exercise Group

	Age (years)	Height (cm)	Weight (kg)	BMI
mean	24.9	172.8	69.0	23.0
sd	1.9	7.6	9.7	2.2

**Table 3:** Demographics of the Heat Immediate Group After Exercise Group

	Age (years)	Height (cm)	Weight (kg)	BMI
mean	26.1	166.1	67.0	24.2
sd	2.6	10.1	12.6	3.5

**Table 4:** Demographics of the Moist Heat After 24 Hours Group

	Age (years)	Height (cm)	Weight (kg)	BMI
mean	26.3	167.4	66.7	23.6
sd	2.8	7.6	13.7	3.4

**Table 5:** Demographics of the Heat 24 Hours After Exercise Group

	Age (years)	Height (cm)	Weight (kg)	BMI
mean	24.9	166.3	62.6	22.5
sd	3.0	10.6	15.5	4.0

## Methods

### Measurement of muscle strength

Muscle strength was measured with the subjects in the seated position with their leg held dependent. An ankle strap was connected from just above the ankle to a stainless steel bar. The bar contained 4 strain gauges placed on opposite sides of the bar. When the bar was bent, the strain gauges, arranged as a Wheatstone bridge, were deformed and an electrical output was provided to a BioPac (BioPac Systems, Goleta, CA) system DAC100 bioelectric amplifier module. The signal was amplified 5,000 times and then digitized through a BioPac MP150 analog to digital converter at a resolution of 24 bits and a frequency of 1,000 samples per second, and stored digitally for later analysis (Acknowledge 4.1 software, BioPac Inc., Goleta, CA). Maximum isometric muscle contraction was measured twice as a 3 second maximal effort and at least 1 minute was allowed between contractions. The average of the two isometric contractions was used in the data analysis as the subject’s maximum strength.

### Exercise

To induce DOMS, the subjects accomplished squats for 5 minutes. They repeated the exercise after 3 minutes of rest for a total of 3 sets of squats. The depth of each squat was at 90° or below.

### Subjective pain measurement

A 10 cm horizontal line was drawn across a piece of paper. One end was marked “pain free” and the other “very, very sore”. The subject was asked to place a vertical slash across the line where appropriate. The location of the slash was converted into a number, where 0 indicated pain free and 10 indicated very, very sore. Only one visual analog pain scale was printed on a single sheet of paper.

### Force to flex and extend the knee (FK)

The force to flex and extend the knee was measured over the range of 90 to 125 degrees. The subject was in the seated position with the leg dependent and supported off of the floor. A linear actuator was connected through an ankle strap to passively move the knee through 35 degrees of flexion. The rate of movement was 6 degrees per second. The knee was flexed and then extended and the force was measured in each direction by 4 strain gauges. The bridge output was amplified and conditioned with a DAC100 strain gauge amplifier with a gain of 500 (BioPac Systems, Goleta, CA). The amplified output was digitized at 2000 Hertz with a resolution of 24 bits on an MP150 BioPac data acquisition system (BioPac Systems, Goleta, CA). A goniometer measured the angle of the knee.

### Measurement of skin resistance

Electrical resistance was measured with a zone finder from Mettler Electronics (Anaheim, CA). It supplied a constant voltage between two probes to measure the micro current between the electrodes. The two probes were tipped with cotton pads and mounted in housing where the distance between probes could be changed, and the force of each probe on the skin could be measured on two separate force gauges. Due to the angle of the probes, pressure caused the skin between the probes to stretch. During each test, the cotton pads on the probes, soaked with 0.9% saline, were placed onto the subject so that equal pressure was applied on each probe, as measured by each force gauge. The skin was first cleaned to minimize the effects of dirt, sweat, or anything else on the surface of the subject. Skin current was measured at 9 locations above the quadriceps in each leg and the data shown in the figures is the average of 18 measurements.

### Heat therapy

Heat was applied by placing either 1 ThermaCare heat wrap or moist heat wrap on each leg centered over the quadriceps and lying longitudinally over the muscle.

### Procedures

On each day, subjects entered the room and relaxed in a thermally neutral environment for 20 minutes. Measurements such as leg strength, tissue resistance, analogue visual pain scales, and force to move the leg were recorded. These data were collected on a Monday, exercise was accomplished on Tuesday and then measurements were taken again on Wednesday, Thursday and Friday. This study consisted of five groups. The control group did not receive any modality. The experimental groups were divided into four groups, one group had ThermaCare heat wraps applied immediately after exercise and another group applied ThermaCare heat wraps 24 hours post exercise. The other two groups either had moist heat applied immediately after exercise or 24 hours post exercise. Heat wraps were placed on the long axis of the quadriceps bilaterally for 8 hours for dry heat and for 2 hours for moist heat.

### Data analysis

Statistical analysis involved the calculation of means and standard deviations and ANOVA. The level of significance was P < 0.05.

## Results

### Strength

**Figure 1:**
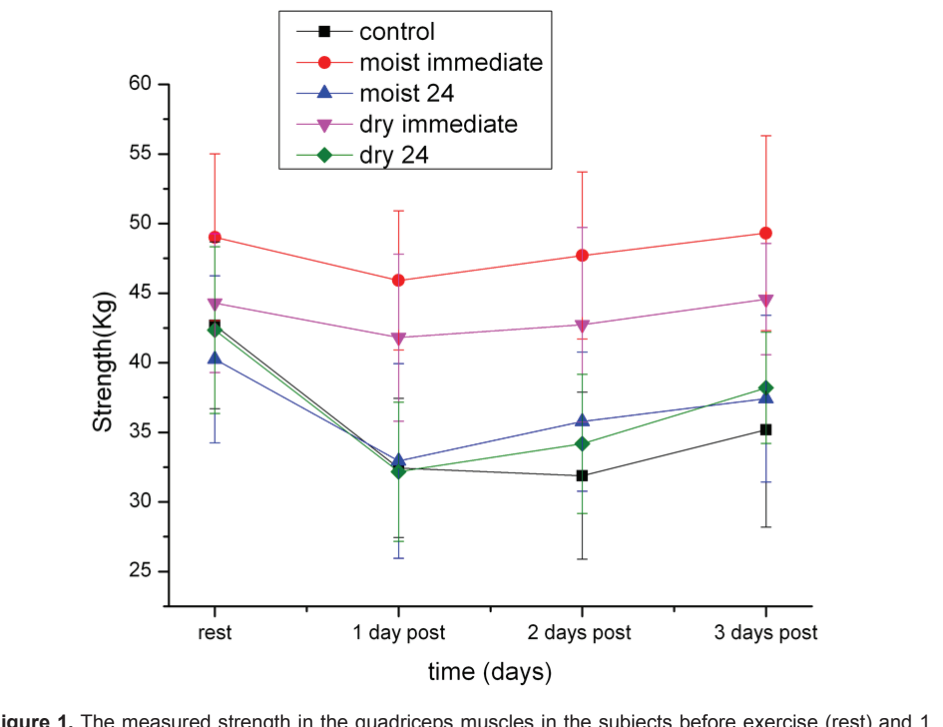
The measured strength in the quadriceps muscles in the subjects before exercise (rest) and 1, 2 and 3 days post exercise. Each point is the mean of 20 subjects ± the standard deviation for controls and after dry heat and moist heat.

As shown in Figure 1, control group showed a significant reduction in quadriceps strength on the 1st day following exercise. This significant reduction (P < 0.01) was 23.8% less than the resting (pre exercise) strength. Both the dry heat immediate and moist heat immediate groups also had a significant reduction in muscle strength on the 1st post exercise day of 4.3% and there was no significant difference between the groups for both moist and dry heat application on this day. The heat immediate groups (moist and dry) recovered by the second day post exercise to the pre exercise strength. The difference between the heat immediate and moist heat immediate groups was significantly higher the 1st, 2nd and 3rd day post exercise than the strength of the control subjects (P < 0.01).

When moist heat or dry heat was used 24 hours after exercise, the data showed that there was no significant difference in quadriceps strength between these groups and the control group (P > 0.05, ANOVA).

### Force to passively move the leg

**Figure 2:**
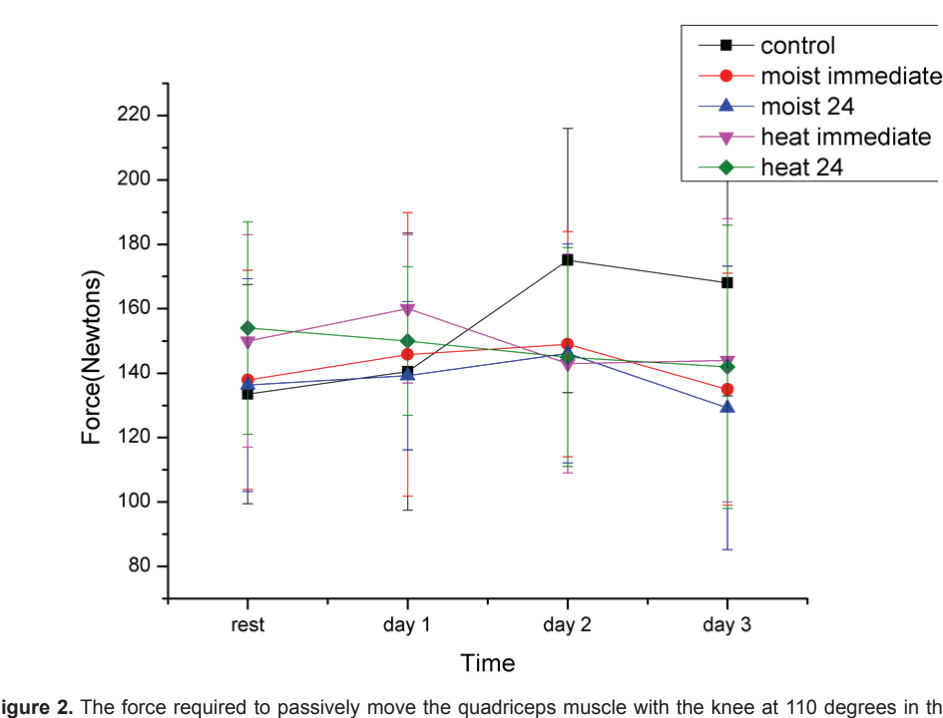
The force required to passively move the quadriceps muscle with the knee at 110 degrees in the subjects before exercise (rest) and 1, 2 and 3 days post exercise. Each point is the mean of 20 subjects ± the standard deviation for the dry and moist heat series.

The force needed to flex the knee was measured from the knee at 90 to 125 degrees. Figure 2 shows the force measured at 110 degrees of knee flexion. This measuring point was used since moving inertia was achieved after movement initiation. At this point, there were some differences in the force to move the leg depending on the leg length and girth of the leg from one individual to the next. Therefore, in this figure, all of the data was normalized in terms of the force to flex the knee before the exercise in each subject. Our results revealed that there was no significant difference in the force to flex the leg one day after the exercise bout between any of the 5 groups of subjects (P > 0.05). In the group that had dry heat applied immediately and 24 hours after exercise, force stayed statistically constant over the next 2 days (P > 0.05 ANOVA). For the group that had no heat, force to move the leg increased significantly on the second and third day (P < 0.01). For the groups that had moist heat applied 24 hours after the exercise, force was the same on the first, second and third days post exercise (P > 0.05).

**Figure 3:**
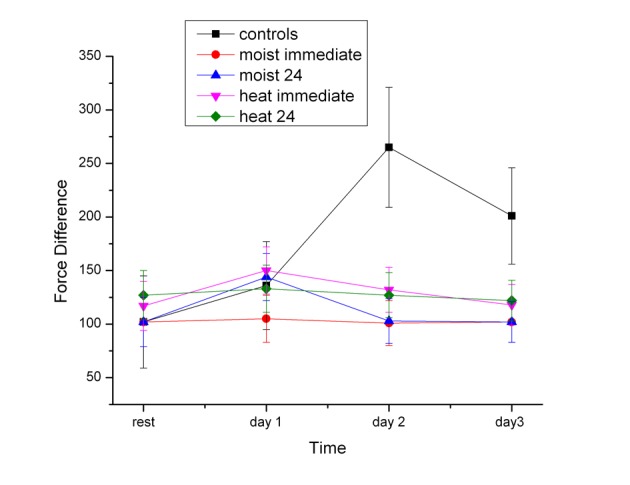
The force required to passively move the quadriceps muscle with the knee at 110 degrees during flexion minus extension force in the subjects before exercise (rest) and 1, 2 and 3 days post exercise. Each point is the mean of 20 subjects ± the standard deviation fir the dry and moist heat series.

Figure 3 shows the hysteresis curve for the same measurement. The forces to flex the knee at the 110 degree point and the force measures during extension at the 110 degree point were different. This is due to stored elastic energy during flexion which is given back during extension and is called hysteresis. As shown in Figure 3, for the 2 groups that received dry heat immediate and 24 hours post exercise and the 2 groups that received moist heat immediately after and 24 hours post exercise, the hysteresis stayed constant over the 4 day period. But for the control group, there was an increase in the difference between the force of flexion and extension that peaked on the 2nd day post exercise which was still significantly higher than rest and at the last day of measurements (P < 0.01).

### Skin current

**Figure 4:**
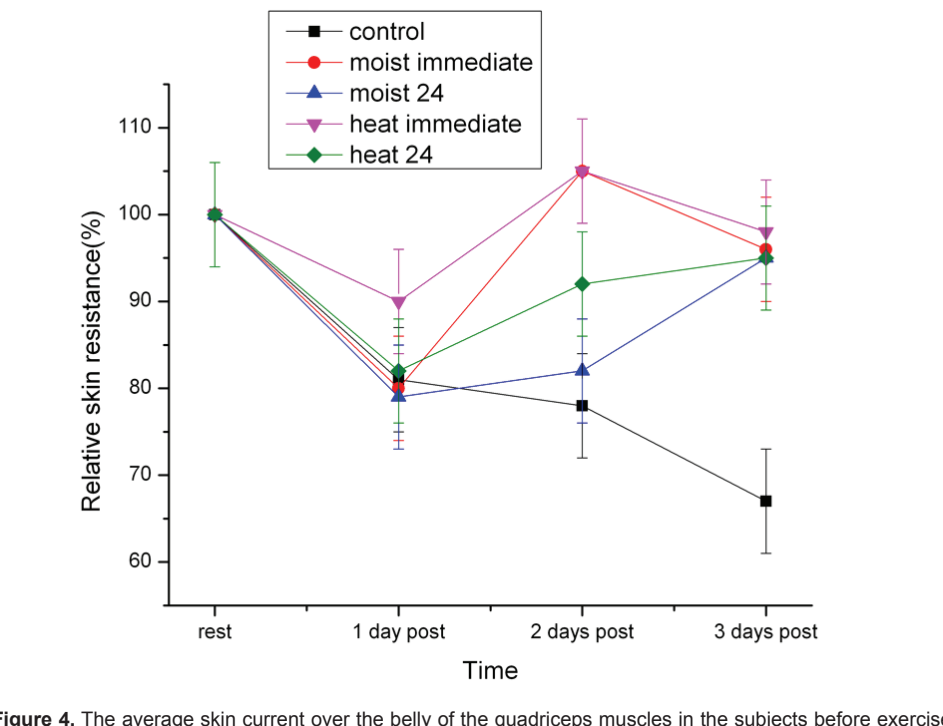
The average skin current over the belly of the quadriceps muscles in the subjects before exercise (rest) and 1, 2 and 3 days post exercise. Each point is the mean of 20 subjects ± the standard deviation for the dry and moist heat series.

The determination of skin current for the quadriceps muscles is shown in Figure 4. There were minor differences in the resting micro current from one subject to the other, perhaps due to differences in subcutaneous fat thicknesses or skin moisture. Therefore, the current was expressed as a percent of the first day’s current as shown in these figures. After the first day, the skin currents were significantly lower in the control groups by day 1 and 2 and by day 3 even lower (P < 0.01). By the second day, the heat immediate group and heat 24 hour groups returned to the pre exercise values (P > 0.05). After moist heat, there was no significant difference between both moist heat and the control group (P > 0.05).

### Analog visual pain scale

**Figure 5:**
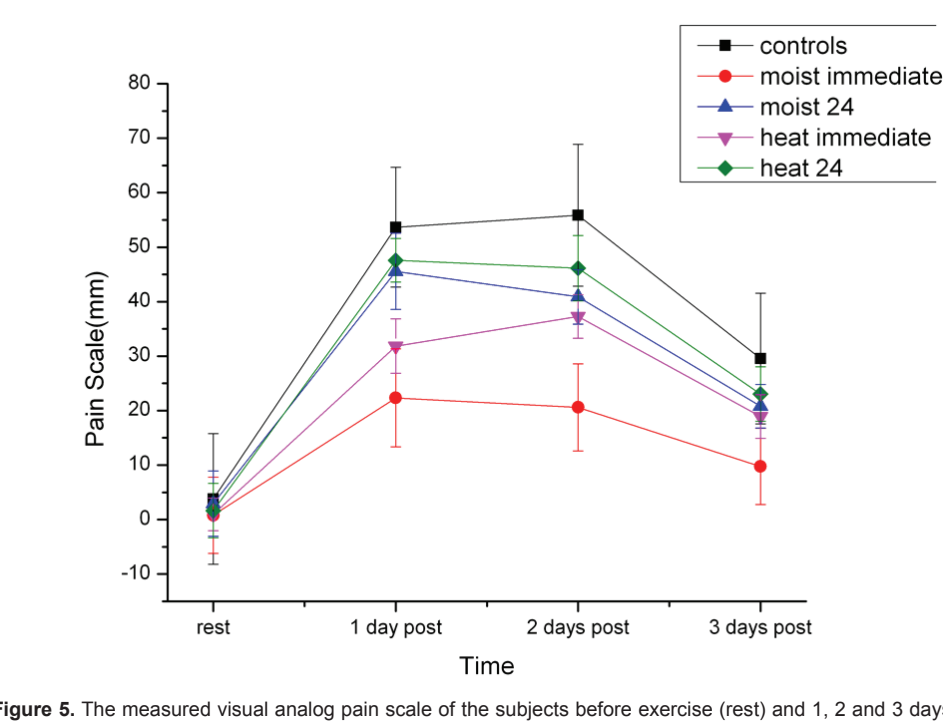
The measured visual analog pain scale of the subjects before exercise (rest) and 1, 2 and 3 days post exercise. Each point is the mean of 20 subjects ± the standard deviation for the dry and moist heat series.

The results of the analog visual pain scale determination are shown in Figure 5. As can be seen in Figure 5, all subjects showed an increase in pain the first day after the exercise. The pain peaked by 2 days post exercise. The least pain was felt 1 day post exercise in the moist heat immediate group. There was no statistical difference between the heat 24, the cold 24 hours the control group post exercise (P > 0.05).

## Discussion

Most clinicians feel that moist heat penetrates deep tissues better than dry heat for warming [30]. This is supported by research examining heat transfer from various types of heat modalities from skin to subcutaneous tissues [15]. Moist heat modalities transfer heat much faster than do dry heat modalities and research shows that they cause much faster heat penetration than dry heat [15, 17, 31]. Even air with high humidity transfers heat faster than dry air [16]. But it is not just the type of heat but the duration as well that affects heat transfer into deep tissues.

For example, contrast baths use warm and cold water immersion that alternates within minutes in warm and cold baths [32]. While this changes skin temperature, there is no evidence that it penetrates into deep tissues [3, 4, 33]. Whirlpool heat penetrates quickly, but is used for only a short duration as are hydrocolator heat packs which provide moist heat but are left on for less than 20 minutes due to their high temperature [34]. Someone with thick subcutaneous fat will therefore only see a small difference in deep tissue temperatures with these modalities [17]. To penetrate deep into tissue, lower temperature and long duration heat packs are often used. Long duration heat products (for example, chemical dry heat) offer the advantage of being safer and can be left on for hours to warm deep tissue and provide increased circulation and pain relief [2]. But the increase in tissue temperature is slow as is the onset of pain relief. Chemical moist heat lasts for a shorter duration than dry chemical heat packs, lasting between 30 minutes and two hours [15]. This study was conducted to determine if moist heat (2 hours), due to its faster heat penetration, would work as well as dry heat wraps (8 hours).

The results can be divided into pain management and pathological changes in muscle after exercise. Muscle strength, a measure of muscle damage, was preserved after exercise equally by heat immediately or moist heat immediately after exercise. But if either modality was used 24 hours after exercise, they had little effect on recovery of strength. Another measure of damage is the force to flex the knee and the difference in force between flexion and extension. This difference is called hysteresis and is related to changes in tissue stiffness [35]. For the force to flex the knee, moist heat and dry heat after exercise and 24 hours later both helped reduce damage as was the case for hysteresis, showing that even though strength was not protected, if heat and moist heat were used 24 hours after exercise, structural damage to the elastic components in muscle or tendon appeared to be eliminated. This offers an interesting dichotomy. Some argue that this is due to soft tissue and to muscle damage [36].

Since strength was reduced when heat was applied at 24 hours, there was damage to the muscle fibers unresolved by heat. But since elasticity was maintained if heat was applied at 24 hours, then a logical conclusion is that heat after 24 hours allowed the healing of tendon and connective tissue so that elasticity was retained but did not help the muscle fibers themselves. This is also shown in the skin resistance data. Here moist heat immediately after exercise was just as effective as dry heat applied immediately and 1 day post exercise. But for pain, moist heat immediately after exercise was more effective than dry heat just after exercise. If either modality was applied 24 hours post exercise, the effects were minimal on pain.

The greater preservation of strength with heat is probably related to increased blood flow in deep tissues [37]. This would increase metabolism and wash away metabolic products that would increase damage to the tissues. Metabolism in tissue doubles for every 2 degree centigrade increase in tissue temperature, allowing for greater healing [37]. Normal shell tissues such as the quadriceps are as much as 5 degrees C less than core temperature [38, 39]. Thus increasing temperature would as much as quadruple metabolism. It is normal for muscle blood flow to increase after exercise due to inflammation and injury [34, 40] such as with delayed onset muscle soreness. But the slow application of heat should cause blood flow to stay elevated to a much higher level and for a longer period of time and allow for better healing of the tissues damaged by DOMS. Pain relief was also seen with moist and dry heat. This is probably due to both faster healing and also gating of pain by skin temperature sensitive ion channels blocking deep pain.

The results of these experiments then show that in spite of the fact that moist heat was only applied for ¼ the duration of dry heat, it was just as effective if not more effective in reducing pain and muscle damage after exercise.

## References

[1] Fleetwood-Walker SM, Proudfoot CW, Garry EM, Allchorne A, Vinuela-Fernandez I, Mitchell R (2007). Cold comfort pharm. Trends Pharmacol Sci.

[2] Mayer JM, Mooney V, Matheson LN, Erasala GN, Verna JL, Udermann BE, Leggett S (2006). Continuous low-level heat wrap therapy for the prevention and early phase treatment of delayed-onset muscle soreness of the low back: a randomized controlled trial. Arch Phys Med Rehabil.

[3] Petrofsky J, Lohman E, 3rd, Lee S, de la Cuesta Z, Labial L, Iouciulescu R, Moseley B (2007). Effects of contrast baths on skin blood flow on the dorsal and plantar foot in people with type 2 diabetes and age-matched controls. Physiother Theory Pract.

[4] Petrofsky JS, Lohman E, 3rd, Lee S, de la Cuesta Z, Labial L, Iouciulescu R, Moseley B (2006). The influence of alterations in room temperature on skin blood flow during contrast baths in patients with diabetes. Med Sci Monit.

[5] Peralvarez-Marin A, Donate-Macian P, Gaudet R (2013). What do we know about the transient receptor potential vanilloid 2 (TRPV2) ion channel?. FEBS J.

[6] Krakow D, Vriens J, Camacho N, Luong P, Deixler H, Funari TL, Bacino CA (2009). Mutations in the gene encoding the calcium-permeable ion channel TRPV4 produce spondylometaphyseal dysplasia, Kozlowski type and metatropic dysplasia. Am J Hum Genet.

[7] Vriens J, Appendino G, Nilius B (2009). Pharmacology of vanilloid transient receptor potential cation channels. Mol Pharmacol.

[8] Chao CC, Hsieh SC, Tseng MT, Chang YC, Hsieh ST (2008). Patterns of contact heat evoked potentials (CHEP) in neuropathy with skin denervation: correlation of CHEP amplitude with intraepidermal nerve fiber density. Clin Neurophysiol.

[9] Farage MA, Miller KW, Maibach HI (2010). Textbook of Aging Skin.

[10] Wetsel WC (2011). Sensing hot and cold with TRP channels. Int J Hyperthermia.

[11] Burnstock G (2013). Purinergic mechanisms and pain-An update. Eur J Pharmacol.

[12] Brederson JD, Kym PR, Szallasi A (2013). Targeting TRP channels for pain relief. Eur J Pharmacol.

[13] Verrill P (1990). Does the gate theory of pain supplant all others?. Br J Hosp Med.

[14] Petrofsky J, Paluso D, Anderson D, Swan K, Yim JE, Murugesan V, Chindam T (2011). The contribution of skin blood flow in warming the skin after the application of local heat; the duality of the Pennes heat equation. Med Eng Phys.

[15] Petrofsky J, Bains G, Prowse M, Gunda S, Berk L, Raju C, Ethiraju G (2009). Dry heat, moist heat and body fat: are heating modalities really effective in people who are overweight?. J Med Eng Technol.

[16] Petrofsky J, Bains G, Prowse M, Gunda S, Berk L, Raju C, Ethiraju G (2009). Does skin moisture influence the blood flow response to local heat. A re-evaluation of the Pennes model?. J Med Eng Technol.

[17] Petrofsky JS, Laymon M (2009). Heat transfer to deep tissue: the effect of body fat and heating modality. J Med Eng Technol.

[18] Petrofsky JS, Lawson D, Suh HJ, Rossi C, Zapata K, Broadwell E, Littleton L (2007). The influence of local versus global heat on the healing of chronic wounds in patients with diabetes. Diabetes Technol Ther.

[19] Song QJ, Li YJ, Deng HW (1999). Early and delayed cardioprotection by heat stress is mediated by calcitonin gene-related peptide. Naunyn Schmiedebergs Arch Pharmacol.

[20] Goldsmith PC, Leslie TA, Hayes NA, Levell NJ, Dowd PM, Foreman JC (1996). Inhibitors of nitric oxide synthase in human skin. J Invest Dermatol.

[21] Charkoudian N (2003). Skin blood flow in adult human thermoregulation: how it works, when it does not, and why. Mayo Clin Proc.

[22] Charkoudian N (2010). Mechanisms and modifiers of reflex induced cutaneous vasodilation and vasoconstriction in humans. J Appl Physiol.

[23] Rabini A, Piazzini DB, Tancredi G, Foti C, Milano G, Ronconi G, Specchia A (2012). Deep heating therapy via microwave diathermy relieves pain and improves physical function in patients with knee osteoarthritis: a double-blind randomized clinical trial. Eur J Phys Rehabil Med.

[24] Rabini A, Piazzini DB, Bertolini C, Deriu L, Saccomanno MF, Santagada DA, Sgadari A (2012). Effects of local microwave diathermy on shoulder pain and function in patients with rotator cuff tendinopathy in comparison to subacromial corticosteroid injections: a single-blind randomized trial. J Orthop Sports Phys Ther.

[25] Guild DG (2012). Mechanical therapy for low back pain. Prim Care.

[26] Petrofsky J, Berk L, Alshammari F, Lee H, Hamdan A, Yim JE, Patel D (2012). The effect of moist air on skin blood flow and temperature in subjects with and without diabetes. Diabetes Technol Ther.

[27] Petrofsky JS (2010). A device to measure heat flow through the skin in people with diabetes. Diabetes Technol Ther.

[28] Draper DO, Hopkins TJ (2008). Increased intramuscular and intracapsular temperature via ThermaCare Knee Wrap application. Med Sci Monit.

[29] Trowbridge CA, Draper DO, Feland JB, Jutte LS, Eggett DL (2004). Paraspinal musculature and skin temperature changes: comparing the Thermacare HeatWrap, the Johnson & Johnson Back Plaster, and the ABC Warme-Pflaster. J Orthop Sports Phys Ther.

[30] Chou SY, Liu HE (2008). Comparison of effectiveness between moist and dry cryotherapy in reducing discomfort after orthognathic surgery. J Clin Nurs.

[31] McLellan K, Petrofsky JS, Bains G, Zimmerman G, Prowse M, Lee S (2009). The effects of skin moisture and subcutaneous fat thickness on the ability of the skin to dissipate heat in young and old subjects, with and without diabetes, at three environmental room temperatures. Med Eng Phys.

[32] Higgins T, Cameron M, Climstein M (2012). Evaluation of passive recovery, cold water immersion, and contrast baths for recovery, as measured by game performances markers, between two simulated games of rugby union. J Strength Cond Res.

[33] Breger Stanton DE, Lazaro R, Macdermid JC (2009). A systematic review of the effectiveness of contrast baths. J Hand Ther.

[34] Petrofsky J, Batt J, Bollinger JN, Jensen MC, Maru EH, Al-Nakhli HH (2011). Comparison of different heat modalities for treating delayed-onset muscle soreness in people with diabetes. Diabetes Technol Ther.

[35] Nordez A, McNair PJ, Casari P, Cornu C (2009). The effect of angular velocity and cycle on the dissipative properties of the knee during passive cyclic stretching: a matter of viscosity or solid friction. Clin Biomech (Bristol, Avon).

[36] Lewis PB, Ruby D, Bush-Joseph CA (2012). Muscle soreness and delayed-onset muscle soreness. Clin Sports Med.

[37] Clarke RS, Hellon RF, Lind AR (1957). The influence of muscle temperature on sustained contractions to fatigue. J Physiol.

[38] Rowell LB, Murray JA, Brengelmann GL, Kraning KK, 2nd (1969). Human cardiovascular adjustments to rapid changes in skin temperature during exercise. Circ Res.

[39] Rowell LB, Brengelmann GL, Murray JA, Kraning KK, 2nd, Kusumi F (1969). Human metabolic responses to hyperthermia during mild to maximal exercise. J Appl Physiol.

[40] Al-Nakhli HH, Petrofsky JS, Laymon MS, Berk LS (2012). The use of thermal infra-red imaging to detect delayed onset muscle soreness. J Vis Exp.

